# So many choices, so little time: Food preference and movement vary with the landscape of fear

**DOI:** 10.1002/ece3.10330

**Published:** 2023-07-26

**Authors:** Clara Mendes Ferreira, Melanie Dammhahn, Jana A. Eccard

**Affiliations:** ^1^ Animal Ecology, Institute for Biochemistry and Biology University of Potsdam Potsdam Germany; ^2^ Behavioural Biology, Institute for Neuro‐ and Behavioural Biology University of Münster Münster Germany

**Keywords:** foraging behavior, functional traits, giving‐up density, *Myodes glareolus*, perceived predation risk

## Abstract

Spatial and temporal variation in perceived predation risk is an important determinant of movement and foraging activity of animals. Foraging in this landscape of fear, individuals need to decide where and when to move, and what resources to choose. Foraging theory predicts the outcome of these decisions based on energetic trade‐offs, but complex interactions between perceived predation risk and preferences of foragers for certain functional traits of their resources are rarely considered. Here, we studied the interactive effects of perceived predation risk on food trait preferences and foraging behavior in bank voles (*Myodes glareolus*) in experimental landscapes. Individuals (*n* = 19) were subjected for periods of 24 h to two extreme, risk‐uniform landscapes (either risky or safe), containing 25 discrete food patches, filled with seeds of four plant species in even amounts. Seeds varied in functional traits: size, nutrients, and shape. We evaluated whether and how risk modifies forager preference for functional traits. We also investigated whether perceived risk and distance from shelter affected giving‐up density (GUD), time in patches, and number of patch visits. In safe landscapes, individuals increased time spent in patches, lowered GUD and visited distant patches more often compared to risky landscapes. Individuals preferred bigger seeds independent of risk, but in the safe treatment they preferred fat‐rich over carb‐rich seeds. Thus, higher densities of resource levels remained in risky landscapes, while in safe landscapes resource density was lower and less diverse due to selective foraging. Our results suggest that the interaction of perceived risk and dietary preference adds an additional layer to the cascading effects of a landscape of fear which affects biodiversity at resource level.

## Graphical abstract

Animals need to make a lot of decisions when foraging on landscapes of fear, but complex interactions between perceived predation risk and dietary preferences are seldom studied. We studied the interaction of perceived predation risk, food trait preference and movement in experimental landscapes, and found that risky conditions lowered foraging time and density of resources. Individuals were opportunistic when feeding regardless of risk, but changed preference for certain food items when conditions were safe, adding an additional layer to cascading effects of landscapes of fear.

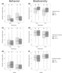

## 1 INTRODUCTION

Foraging is a crucial activity for animals and incurs a variety of decisions (Stephens, 2008) on the location, the balance of metabolic costs, missed opportunity costs and energetic gains, as well as patch leaving decisions (e.g., Brown, 1988; Sih, 1994; Stephens et al., 2007). Furthermore, predation risk is an important determinant of foraging decisions, with foragers adjusting their activities to the spatiotemporal variation of their perception of predation risk, that is, landscape of fear (Gaynor et al., 2019; Laundré et al., 2014). While direct cues from predators can be a strong indicator of mortality risk (Mayer et al., 2020; Sivy et al., 2011), prey species often rely on indirect cues to map their landscape of fear, such as ground cover or illumination (e.g., Mella et al., 2014; Orrock et al., 2004). Based on their landscape of fear, individuals must decide on how much time they allocate to feeding or to risk avoidance (Altendorf et al., 2001; Brown & Kotler, 2004), therefore, there is a trade‐off between the gains of foraging (Charnov, 1976; Stephens & Krebs, 1986) against the risk of mortality (Lima & Dill, 1990).

Important determinants of energy gain for the forager are functional traits of the resources that are consumed, that is, the characteristics which increase the fitness of resource species (Adler et al., 2013). These often entail attributes that are preferred by foragers (Lantová & Lanta, 2009; Salgado‐Luarte & Gianoli, 2012), especially under optimal foraging theory (i.e., foragers will maximize energetic gains with the more profitable resources in the least time possible; Charnov, 1976; Sih & Christensen, 2001). For example, in plants one common functional trait is energy reserves, and bigger plant seeds have higher germination rates compared to smaller seeds (Jakobsson & Eriksson, 2000). Meanwhile, bigger seeds are also preferred by foragers as they contain more energy per item (Gómez, 2004), even though bigger seeds are also more difficult or time consuming to handle and/or transport (Boone & Mortelliti, 2019; Chang & Zhang, 2014), therefore impacting the final energetic gain or possibly increasing mortality risk by predation with increased patch residency (Lima, 1985; Newman et al., 1988). The nutrient values of each food item might also affect the forager's behavior, with individuals preferring items that are more caloric (Gerber et al., 2004). Therefore, functional traits of resources and dietary preferences of foragers are often interlinked, but as perceived predation risk also affects the forager's behavior, it can alter the choice for certain resource traits. Ultimately, the landscape of fear generates behavioral‐mediated cascading effects that alter the final composition of communities of resource species (Eccard et al., 2022), and foragers can affect the coexistence of plant species (Ferreira et al., 2022; Garb et al., 2000; Stump & Chesson, 2017). To better understand the complex demographic and biodiversity effects of foraging in different landscapes of fear—especially at the community level of the resources—it becomes important to understand forager's dietary preferences for specific functional traits, and how perceived predation risk modifies these preferences.

A forager also has to decide when and where to forage, and this decision is based on the balance between energy gains of feeding against possible mortality risks (Mitchell & Lima, 2002). Metabolic cost of movement itself, either of foraging behavior or the risk during movement and/or feeding, also contribute to the outcome of this decision. Animal movement is then directly tied with energy landscapes, that is, the metabolic costs of movement for animals, both in space and in time, based on the physical properties of the habitats (Gallagher et al., 2017; Masello et al., 2021). Animals tend to choose pathways that minimize metabolic costs, while maximizing the energy gain (e.g., moving to high‐quality food patches; Wilson et al., 2012), which also includes minimizing the costs of potential predation risk (e.g., movement can make the prey itself more visible to predators; Ciuti et al., 2012; Turcotte & Desrochers, 2003). The risk while traveling in a matrix between food patches can affect the duration of travel and, therefore, energetic gains of foraging (Eccard et al., 2020). Thus, movement is affected by three components: potential energy gain of foraging, energetic losses through movement (and foraging) itself, and potential energy loss through predation risk avoidance. To both maximize gain and minimize risk, the forager could forage closer to its shelter or refuge (i.e., safe place to rest), as described in the central‐place foraging theory (foragers first exploit patches near the central location, except if distant patches have high quality and profitable resources; Bakker et al., 2005; Fryxell, 1999, Nilsson et al., 2020; Orians & Pearson, 1979; Schoener, 1979).

Basically, the act of foraging involves many decisions for animals simultaneously, concerning risk taking, nutritional value of resources, and movement. To better understand the process of decision making when foraging in landscapes of fear and biodiverse resources, we need to consider all these processes together. Therefore, we aimed to test the behavioral‐mediated cascading effects on the resource levels caused by differential feeding under different landscapes of fear, and further study how movement and foraging activity are affected by perceived predation risk levels. We use a ground‐dwelling rodent as the study species (bank vole, *Myodes glareolus*). Rodents are widely used in studies regarding landscapes of fear, as one can manipulate their perception of fear easily though manipulating the cover (rodents decrease their foraging activity in low to no cover; e.g., Dammhahn et al., 2022; Eccard & Liesenjohann, 2014; Kotler, 1992). Furthermore, illumination can also be used to manipulate perceived risk, as rodents decrease their activity under light to avoid being highly visible to avian predators (e.g., Clarke, 1983; Hoffmann et al., 2018, 2019; Kotler et al., 1991; Rotics et al., 2011), with bank voles being a suitable species for light manipulation experiments as they present polyphasic activity patterns (Halle, 2006; Ylönen et al., 1988). Perceived predation risk can also be quantified using the giving‐up density (GUD), that is, the density of food resources left in a patch after the individual decides to stop foraging due to decreased gains over the increasing predation risk (Brown, 1988). Like most seed‐eating rodents, bank voles also show preference for specific food functional traits, with size and/or high energetic content being the most preferable traits (Eccard & Ylönen, 2006; Ellingsen et al., 2017; Fischer et al., 2017; Fischer & Türke, 2016). This is in concordance with other rodent species, which also show preference for larger and/or nutrient‐rich seeds (Boone & Mortelliti, 2019; Hou et al., 2021; Kelrick et al., 1986; Mortelliti et al., 2019; Wang & Corlett, 2017), with fat content being the most preferred nutrient. Rodents can also show preference for certain seed shapes as, together with size, some seeds might be more easily handled and transported than others (Muñoz et al., 2012). Under varying predation risk, we expect foragers pursue an optimal foraging strategy, with the effects possibly being more visible in high perceived risk, as foragers spent less time in the patch (lower patch residency) and might try to maximize gains by foraging on the most profitable (bigger and/or most caloric seeds) or easier to handle resources (e.g., circle shaped seeds). However, it is still difficult to disentangle which of these three functional traits actually influences foraging dietary preferences and if perceived predation risk affects these choices, especially choice between size and nutrient value, as these functional traits are often correlated (Wang & Yang, 2014).

Under indoor captive conditions, we introduced bank voles to two risk‐uniform landscapes of fear (safe vs. risky) with discrete patches of uniform initial resource diversities. We hypothesized that:

- Perceived predation risk differences can be confirmed by changes in foraging behavior, as foragers consume less resources, but will also move less and spend less time foraging in a risky landscape compared to a safe landscape;
- Under different perceived predation risk treatments, we expect an interaction of perceived risk and forager's dietary preference for certain functional traits. As foragers become more active in safer conditions, they prioritize one functional trait over others.

We further monitored the spatial distribution of foraging effort, assuming that animals would forage on patches closer to their shelter (central‐place foraging theory; Fryxell, 1999; Orians & Pearson, 1979, Schoener, 1979), and assumed that this concentration of foraging effort would be higher under risky conditions (Eccard & Liesenjohann, 2008).

## 2 MATERIALS AND METHODS

### 2.1 Animal housing and experimental design

We captured 19 individuals (13 females and six males) from wild bank vole populations near Potsdam, Germany (52°26′17.4″ N 13°00′22.4″ E) in October 2018. We used Ugglan live traps (Grahnab Sweden, Special No. 2, with shrew exit) baited the traps with rolled oats and apples. Upon capture, we weighed, sexed, and identified age and reproductive status. Lactating or pregnant females were immediately released at the capture site. The 19 individuals were transferred to the housing room at the laboratory of the Animal Ecology group of the University of Potsdam, and kept singly in standard makrolon cages (Ehret GmbH Germany, Type III: 42 cm × 27 cm × 16 cm), with bedding (wood shaving and hay). Food pellets (ssniff® NM, ssniff® R/M‐H Ered II) and water were provided ad libitum. The room was kept at ca. 20°C and the photoperiod was adjusted to seasonal day length.

**FIGURE 1:**
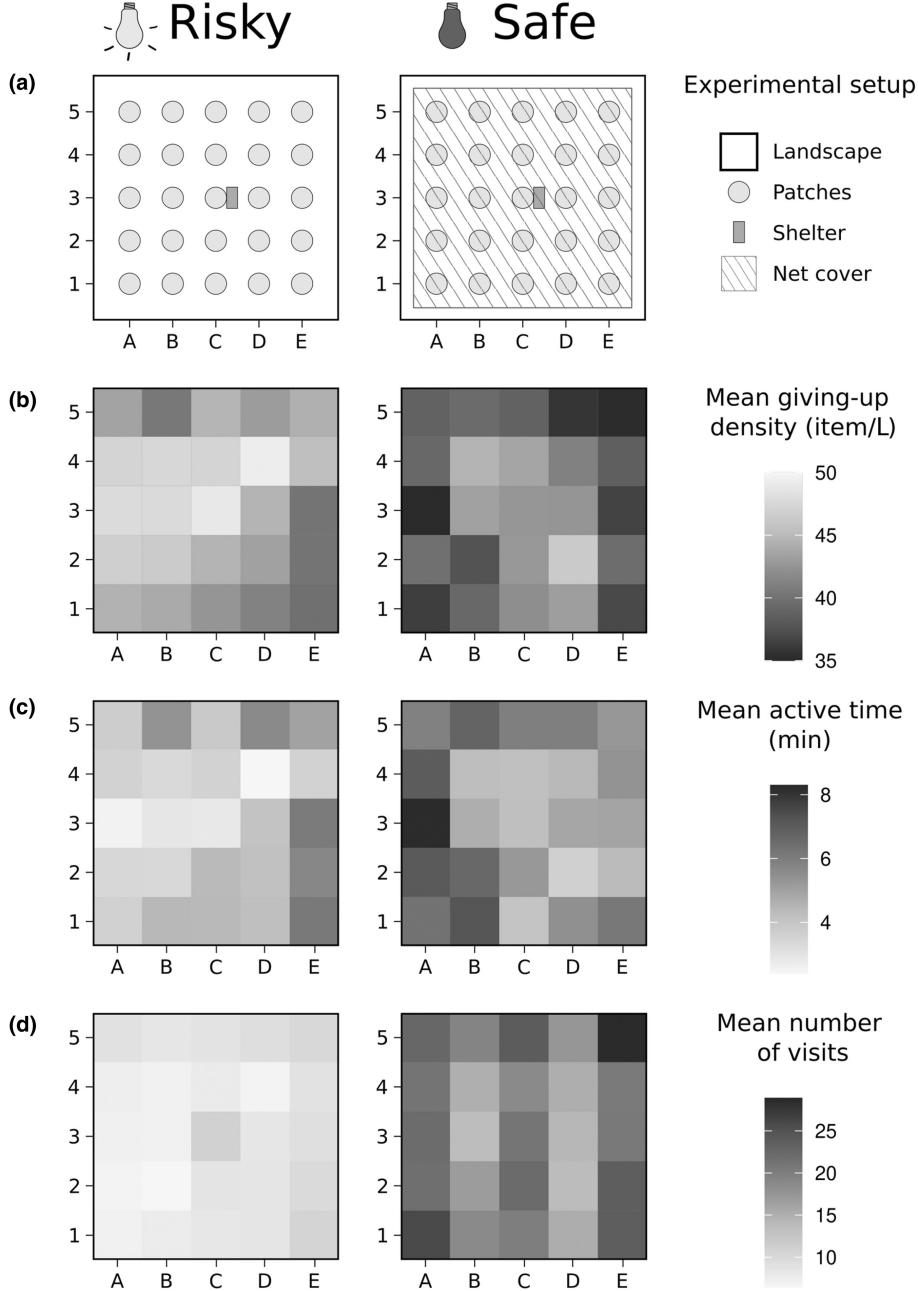
(a) Experimental setup by perceived predation risk treatment (risky—no cover, under light; safe—cover, under dark), with the 25 patches spaces by 50 cm both horizontally (A–E) and vertically (1–5) in the landscape, and with shelter and water in the center (position C3). Greyscale heat‐maps of: (b) mean giving‐up density (GUD), (c) mean time in patch, and (d) mean number of visits at each patch across all 19 animals.

The experiments were done between April and July 2019, with the second treatment being done with at least 2 weeks of separation in between, and occurred in indoor arenas (therein landscapes) of 2.5 m × 3.0 m, surrounded by 0.7 m tall galvanized metal fences. Each individual was placed singly in one arena and four arenas were run in parallel in the same room, with two of them running under light and the other two running under dark with a thick curtain dividing both sides. In the center of each arena, we put a small wooden nest box and a water bottle providing individuals full access to a safe shelter and water ad libitum during the duration of the experiment. Each landscape had a grid of 25 seed trays (therein patches, round plastic bowls of 13 cm diameter and 4.5 cm height) separated by 50 cm (Figure 1a). Each patch was filled with 0.5 L of sand and seeds from four plant species with different functional traits (Table 1), with seven individual seeds each (total of 28 seeds in each patch). Seed quantity was chosen to ensure that individuals would have their minimum food intake needs (average 2.5 g/day; Eccard & Ylönen, 2006; Peacock & Speakman, 2001), and even if foraging activity was lower than expected, this seed quantity would still enable us to quantify the diminishing returns of GUD. We video surveyed the whole landscape continuously during the entire experiment, using an analog HD Dome Camera (ABUS HDCC31500 720p) with a top view of the landscape. Prior to our experiment, all individuals experienced the arenas and setup of patches (containing only millet seeds) twice, once for 4 days ca. 2 weeks before the experiment and once for 3 days just before the experiment. Furthermore, all individuals were familiarized with the four seed types in their home cages over 1 week before the experiment. At the beginning of our experiment, all remaining sand or seeds were removed from the area and the shelter to avoid spillover from the previous experiment.

**TABLE 1:** Characteristics of the seeds of four plant species used as resources in the experiment.

Plant species		Husked	Mass per seed (mg)	Calories per seed (J)	Calories per 100 g (J)	Fat (%)	Total carbohydrates (%)	Seed pairs		
								Size	Nutrients	Shape
Wheat	*Triticum aestivum*	**No**	**39.4**	**536.8**	1362.5	1.8	**60.0**	**Big**	**Carb rich**	Oval
Hemp	*Cannabis sativa*	Yes	**11.9**	**229.7**	**1930.3**	**32.0**	22.0	**Big**	Fat rich	**Circular**
Flaxseed	*Linum usitatissimum*	Yes	7.0	158.2	**2259.4**	**42.5**	2.0	Small	Fat rich	Oval
Millet	*Pennisetum glaucum*	**No**	6.1	91.1	1493.9	3.9	**69.0**	Small	**Carb rich**	**Circular**

*Note*: Nutritional values were obtained from the package nutritional tables. Mass per seed item of each species was obtained by weighting 100 seeds and dividing it by 100. Seeds were grouped into pairs based on similarity in functional traits, indicated by similar font (bold or non‐bold text).

Each individual was tested for 24 h once in each of two different risk uniform levels (landscapes of fear): risky—the perceived predation risk was high, the individuals were kept under light with no cover; and safe—the perceived predation risk was low, the individuals were kept under dark with a net cover (mesh size 1.5 cm × 1.5 cm) over the patches to simulate the effect of vegetation cover brushing on animal's fur. We know from previous experiments that illuminated open areas are perceived as risky (Hoffmann et al., 2019) and areas under net cover are perceived as safe by voles (Eccard et al., 2020; Eccard & Liesenjohann, 2008). A net cover also allowed us to record and track the animal position. By providing extreme levels of uniform risk, we could assess the effects of fear in movement and dietary preference of foragers. We varied the order of risk treatments (therein “order”) across individuals, so that the first treatment was either risky or safe and the second treatment would be the opposite. Due to logistic constrains, four individuals only underwent one of the treatments (two individuals safe, two individuals risky). These individuals were also included in the analyses.

After 24 h, the individuals were removed and transferred back to their home cages. We collected the trays, sieved the sand, and counted the remaining seeds that were still intact (i.e., uneaten). We calculated the overall GUD (across all seed types, i.e., food items), and the seed‐specific GUD by dividing the total number of seeds left in each patch by the 0.5 L of sand. Two patches were removed from the dataset as the contents of the trays were accidentally mixed‐up. Seven patches showed signs of human error (more than seven seeds for each species in the final count), however, we kept them in our analyses as we investigate giving‐up densities, which should be independent of initial fillings.

### 2.2 Data and statistical analyses

Tracking of individual movement was done via the AnimalTracker plugin for ImageJ (Gulyás et al., 2016), using the recorded videos of the experiments (for details, see Appendix S1). Using AnimalTracker, we quantified for each active bout (i.e., animal leaving the shelter), the individual's total active time in the arena (time spent outside the shelter, in second), the cumulative time spent in each patch (measure of patch residency, in second), and the cumulative distance traveled (in cm). Due to the automatic tracking methodology of AnimalTracker (i.e., blob detection), the time and distance were counted as soon as the entire head of the animal was visible outside the shelter or inside the patch. Time and distance traveled were later converted and are reported as minute and meter for easier interpretation. Since cumulative time and distance traveled in a patch were highly correlated (Kendall's correlation coefficients (tau) = 0.79, *p* < .001), we only used cumulative time in a patch for subsequent analyses. We then calculated the number of unique visits per patch, with each visit either being the individual moving from one patch to another, or if the individual left the patch for ≥5 s and returned. This interval was chosen to differentiate events where individuals returned to the shelter (e.g., for caching seeds) from events where individuals very briefly exited the patch, as most of the latter events occurred within 5 s. Repeated visits gave us a spatial measure of risk perception, in which animals would only visit patches repeatedly if they perceived the energetic gains (profitability of a patch) as higher and predation risk as lower.

We built linear mixed‐effects models (LMM) with dependent variables GUD, cumulative time spent in a patch, and number of patch visits. We square rooted the cumulative time and number of visits variables to normalize them. Although number of visits is a count variable, the final number of patch visits had a large range (min–max range: 0–56 visits), so it was treated as a continuous variable. We used treatment (risky or safe), order (first or second), and distance of patch to shelter as fixed effects, as well as all two‐way interactions among these three fixed effects. Models included patch and individual identity as random effects (random intercepts) to control for repeated measures, spatial correlation as well possible individual variation. If patch identity explained zero variance, it was dropped from the model. Before running our models, we visually inspected variation across experimental days (four animals tested simultaneously) and across arenas (arenas being used repeatedly). We also used these as random effects in preliminary analyses. Since these variances appeared homogeneous, we decided not to include them as extra random effects in the model to avoid overfitting models.

The importance of functional traits was investigated by grouping the seeds from the four plant species into pairs that shared at least one functional trait of the seeds (size, nutrient and shape—Table 1). By dividing the seeds according to their properties (big or small, carb rich or fat rich, circular of oval), we could distinguish and disentangle which functional traits were important under different risk conditions, rather than knowing which plant species seed would possibly drive the harvest curves. We used a Poisson log‐linked GLMM to test if the total amount of seeds eaten (dependent variable) varied among seed pairs. Each functional trait grouping was analyzed in a separate model (three models). We also included in the model the independent variable interaction of seed pairs with treatment or order. We removed interactions from the model if they were non‐significant, but always kept treatment, order and trait difference as fixed factors. Models included patch and animal ID as random effects (random intercepts) to control for non‐independence among seeds within the same tray, and differences among individual foragers. If patch ID explained zero variance, it was dropped from the model. If any of the interactions were significant, we did a simple post‐hoc comparison using the least‐square means, comparing among factor levels within levels of the respective other factor. To investigate harvesting dynamics of single seed types, we plotted seed‐specific harvesting functions using polynomial regression fittings of seeds by time spend in a patch.

All analyses were done in R 4.0.4 (R Core Team, 2021), and run using the lm4 package (Bates et al., 2015) and the lsmeans package (Lenth, 2016). For each model, we tested if the removal of different fixed effects improved the model fit based on the Akaike information criterion, starting with the least supported interactions, and report the most parsimonious model. Model fit was evaluated based on residual distribution using qqplots. *p*‐values for LMM and GLMM models were obtained through the R package lmerTest (Kuznetsova et al., 2017). The accepted significance level was set to α < 0.05. To correct for repeated testing in the seed pair analyses, we adjusted the significance level with a Bonferroni correction to α = 0.016.

Animal capture, housing and experiment were done under the permission of the “Landesamt für Umwelt” (reference number: AZ: N 10424), “Landesamt für Arbeitsschutz, Verbraucherschutz und Gesundheit (LAVG)” (reference number: AZ: 2347‐46‐2018) in Brandenburg, Germany, and “Landeshauptstadt Potsdam, Veterinärwesen und Lebensmittelüberwachung” (reference number: AZ 386‐1). The experiment was conducted in accordance with all applicable international, national and/or institutional guidelines for the use of animals.

## 3 RESULTS

**FIGURE 2:**
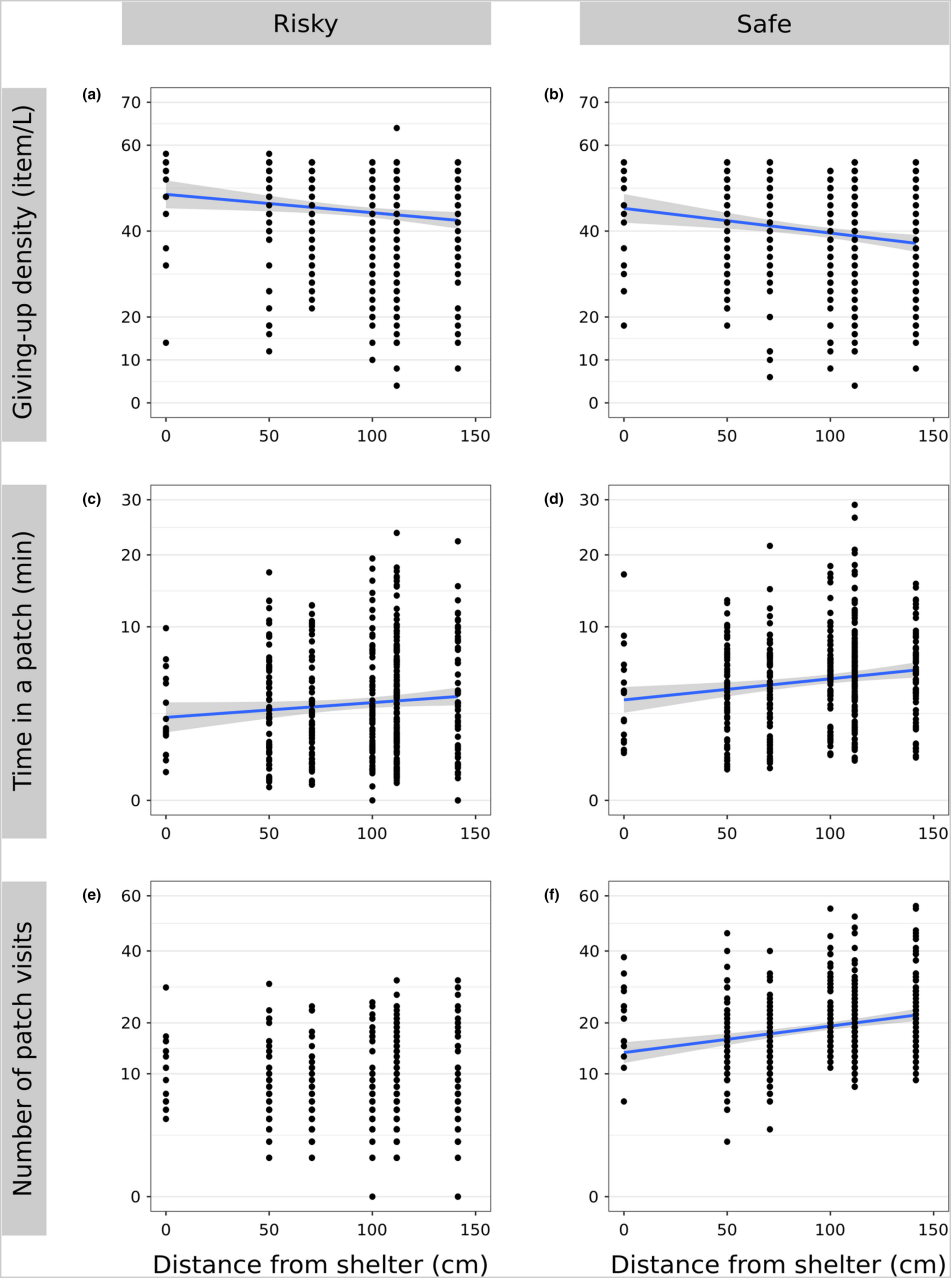
Relation between (a, b) giving‐up density, (c, d) cumulative time spent in a patch (square‐rooted), and (e, f) total number of patch visits (square‐rooted) to the distance from the shelter, between perceived predation risk treatments (risky and safe). The blue trend lines and their 95% confidence intervals (gray) are based on the significant linear models, without random effects for illustration only.

**FIGURE 3:**
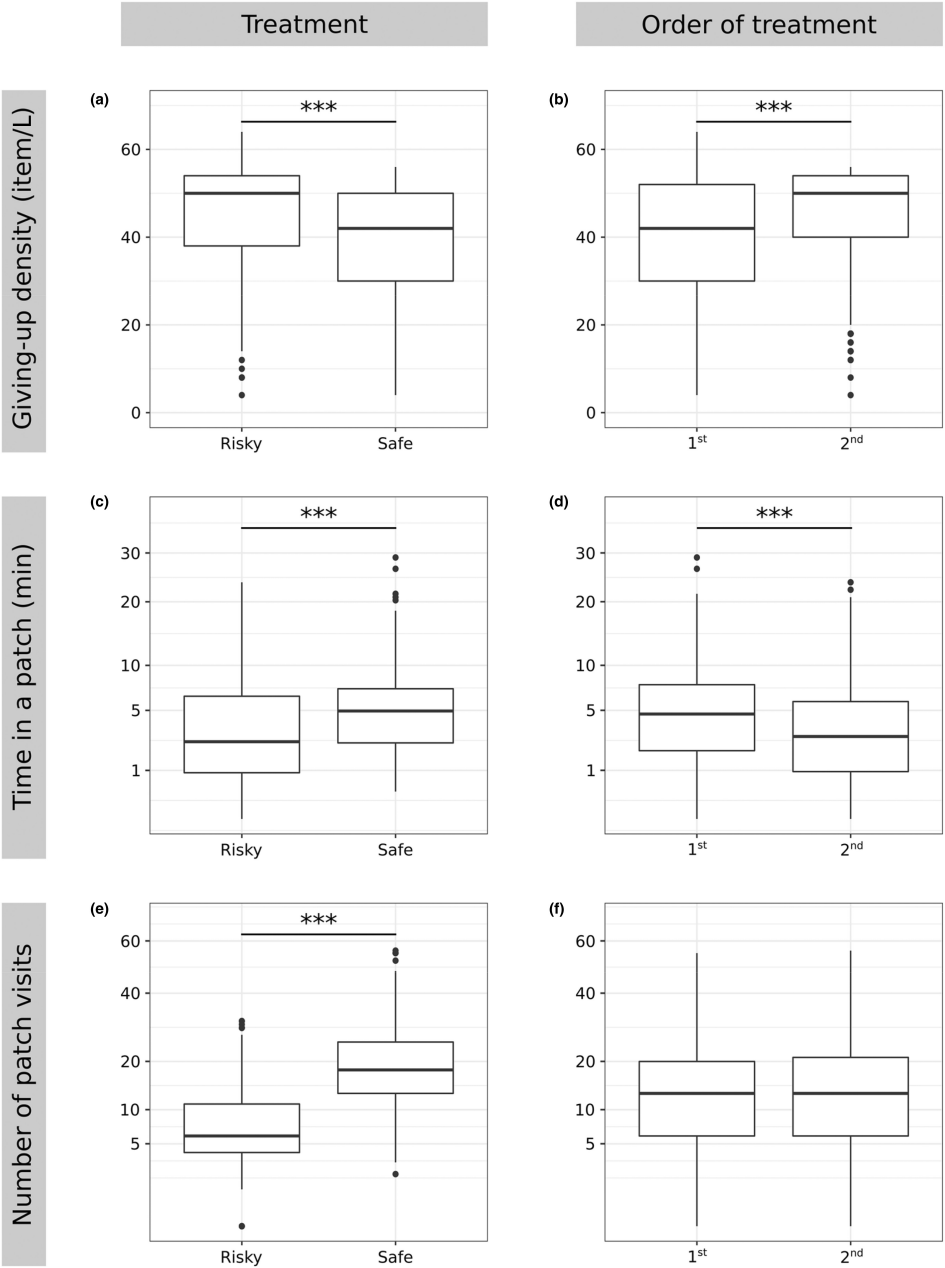
Differences in giving‐up density, cumulative time spent in a patch (square‐rooted), and total number of patch visits (square‐rooted) between perceived predation risk treatments (risky and safe; a, c, e), and between first and second treatment experienced by an individual (order of treatments was randomized; first and second; b, d, f). Significant differences are shown with *** (*p*‐value <.001) above box‐plots.

Over a 24‐h period, individuals traveled 512.49 m ± 326.15 (mean ± standard deviation) and were active for 212 min ± 104. In the safe treatment, GUD was lower and cumulative time spent in a patch was higher compared to the risky treatment (Table 2; Figures 1b,c and 3a,c). On average, individuals moved 744.88 m ± 237.59 in the safe treatment and 280.10 m ± 219.52 in the risky treatment. When subjected to the experiment a second time, GUD was higher and cumulative time spent in a patch was lower (Figure 3b,d), but there were no differences in the number of patch visits (Figure 3f). GUD decreased and cumulative time spent in a patch increased in patches further away from the shelter (Table 2; Figures 1b,c and 2a–d). In the safe treatment the number of patch visits increased with distance from the shelter (Table 2; Figures 1d and 3e), but not in the risky treatment.

**TABLE 2:** Results of final LMMs for the dependent variables of giving‐up density (GUD), cumulative time spent in a patch, and number of patch visits.

	Giving‐up density (item/L)		Time in a patch (square‐root transformed, min)		Number of patch visits (square‐root transformed)	
	*β* ± SE	*p*	*β* ± SE	*p*	*β* ± SE	*p*
Intercept	47.16 ± 1.72		1.60 ± 0.14		2.83 ± 0.24	
Treatment (safe)	**−5.16 ± 0.78**	**<.001**	**0.49 ± 0.06**	**<.001**	**0.91 ± 0.15**	**<.001**
Order (second)	**3.89 ± 0.78**	**<.001**	**−0.39 ± 0.06**	**<.001**	–	
Distance	**−0.05 ± 0.02**	**<.001**	**0.003 ± 0.001**	**<.001**	−0.001 ± 0.002	.75
Treatment*Distance	–		–		**0.007 ± 0.001**	**<.001**
Subset analyses						
Distance (safe)	–		–		**6.83 × 10** ^**−3**^ **± 2.19 × 10** ^**−3**^	**.005**
Distance (risky)	–		–		−5.13 × 10^−4^ ± 1.25 × 10^−3^	.69
Chisq (df)	42.60 (1)		66.30 (1)		1.62 (1)	
*R* ^2^ marginal	0.08		0.11		0.37	
*R* ^2^ conditional	0.27		0.32		0.69	
Patch identity						
Variance	–		–		0.05	
SD	–		–		0.74	
Individual identity						
Variance	30.01		0.20		0.55	
SD	5.48		0.45		0.74	

*Note*: The models included the individual identity as a random effect and some models also included patch identity as a random effect. Categories are given in brackets and compared to reference levels. Shown are estimated fixed effects (*β*), their standard errors (SE), *p*‐values (*p*), Chi‐square (Chisq) with degrees of freedom (df), the *R* ^2^ based on the fixed factors (*R* ^2^ marginal) and based on fixed and random factors (*R* ^2^ conditional), as well variance and standard deviation (SD) of each random effect. All significant relationships are shown in bold font.

**FIGURE 4:**
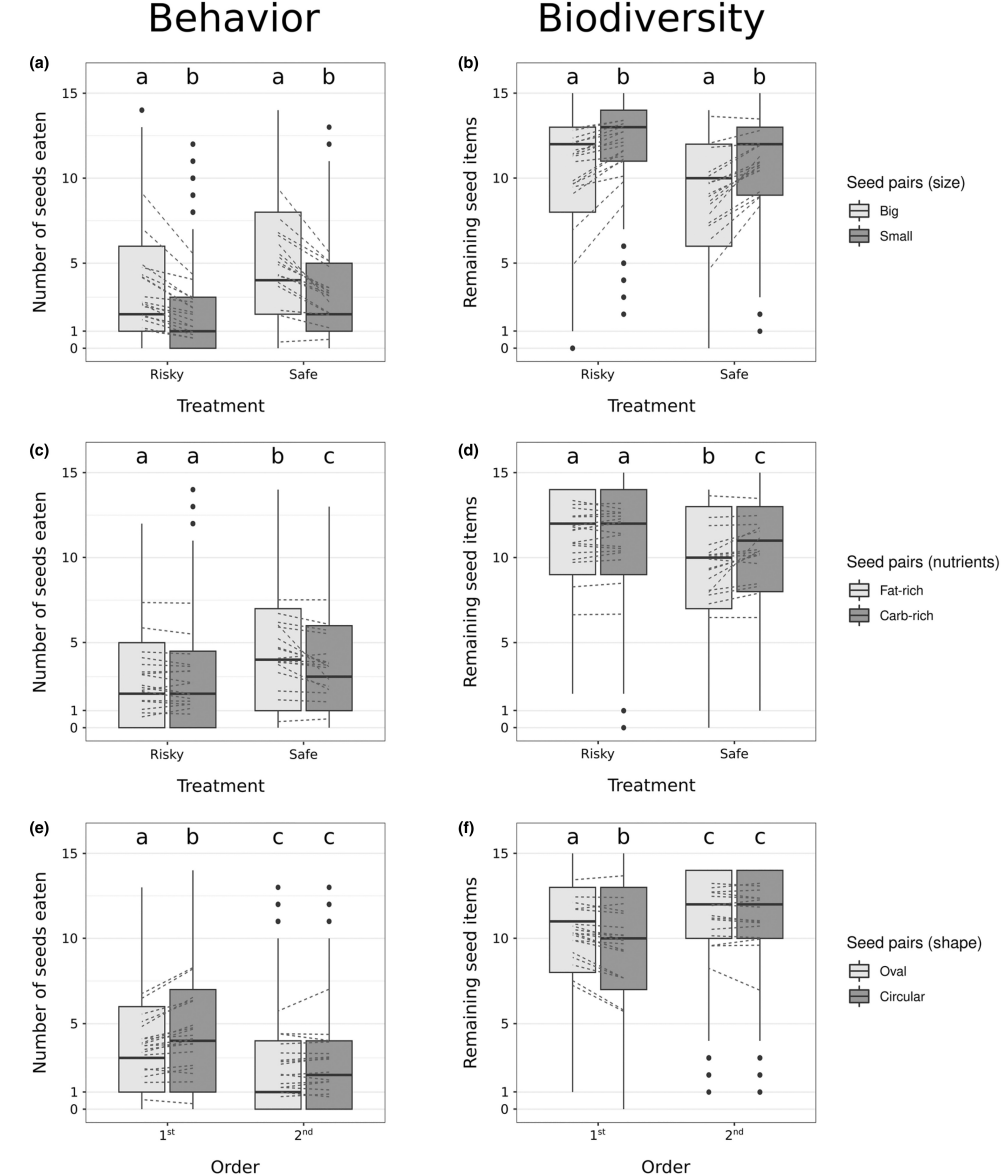
Differences in total number of seeds eaten (forager's “behavior”) or left in the patch (seed community “biodiversity” after foragers give up foraging) for each pair of seeds sharing one functional trait (a) size, (b) nutrient content, and (c) shape between perceived predation risk treatments (risky vs. safe, a and b) and between the first or second treatment experienced by an individual (order of risk treatments was randomized, first and second in c). Dotted lines represent individual responses. Different letters indicate significant group differences based on least‐square means (adjusted with the Tukey method).

**FIGURE 5:**
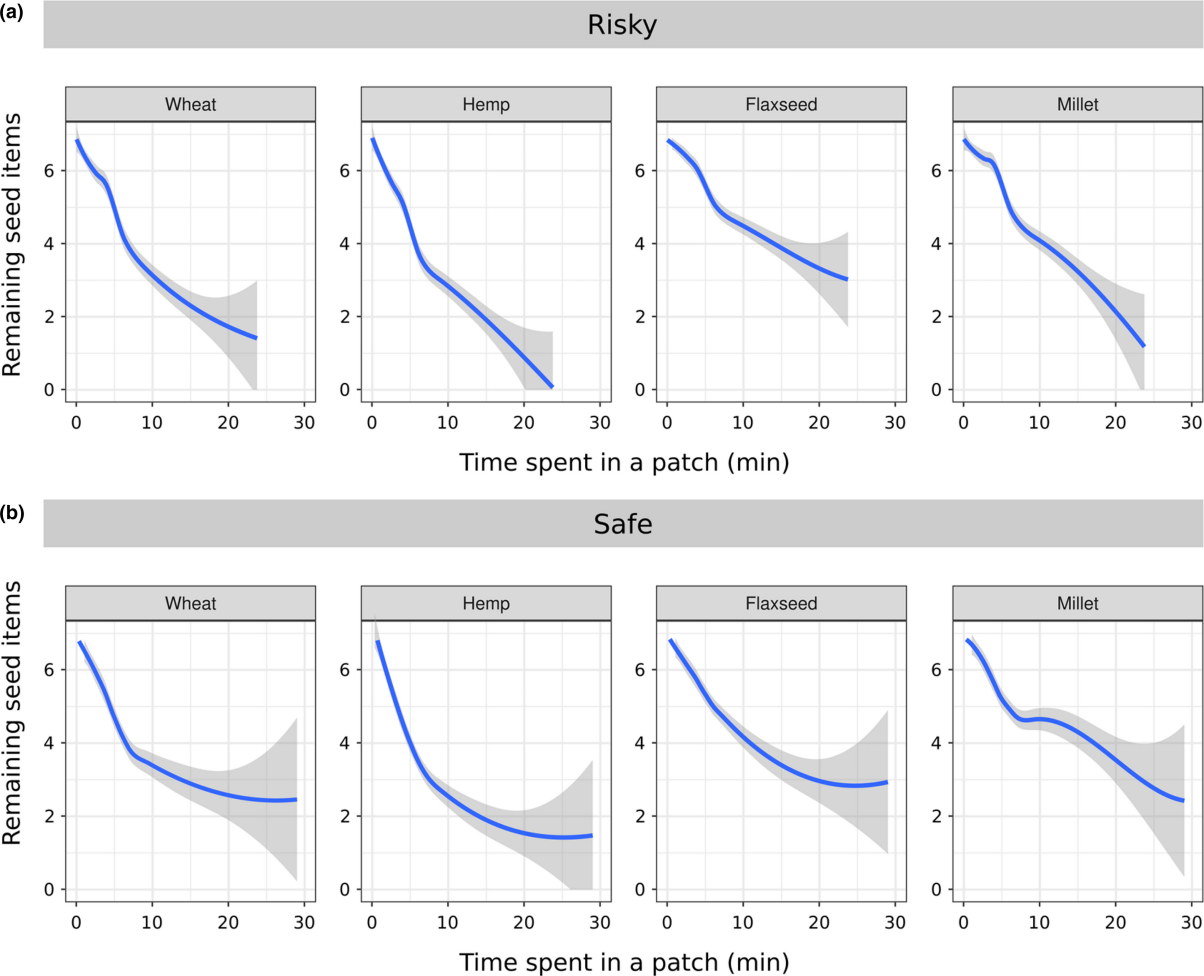
Harvest curves per seed of four plant species (wheat, hemp, flaxseed, and millet, blue lines), showing the remaining seed items and cumulative time spent in a patch between (perceived) predation risk treatments (risky, a, and safe, b). The harvest curves (blue lines) and their 95% confidence intervals (gray) are based on polynomial regression fittings.

Bigger seeds were eaten more than smaller seeds, which were left uneaten more often in the patch (*z*‐ratio = 15.26; *p* < .001; Table 3; Figure 4a,b) regardless of risk treatment. There were tendencies for interactions between seed nutrients and risk treatments, as well as between test order and seed shape (Table 3). In the safe treatment, individuals ate more fat‐rich seeds than carb‐rich seeds, leaving the latter in higher densities in the patch, but there was no effect of nutrients in the risky treatment (Figure 4c,d; Table 4). Individuals ate more seeds in the first run (Tables 2 and 3), and preferred circular seeds over oval in the first run (Figure 4e,f; Table 4). Seed‐specific harvesting curves (Figure 5) suggest that animals reached meaningful GUDs (no further depletion) only in the safe treatment, since an asymptote was reached for each seed type. The bigger seeds (wheat and hemp) reached its value earlier (visual inspection) and were depleted to lower asymptotes than the smaller seeds. Meanwhile, in the risky treatment animals stayed shorter, and asymptotes were not reached for any of the seed types.

**TABLE 3:** Results of final GLMMs on the number of seeds eaten within seed pairs differing in levels of one functional trait: size (big and small), nutrients (carb rich and fat rich) or shape (oval or circular).

	Size		Nutrients		Shape	
	*β* ± SE	*p*	*β* ± SE	*p*	*β* ± SE	*p*
Intercept	1.26 ± 0.11		1.09 ± 0.12		0.99 ± 0.11	
Trait	**−0.43 ± 0.03**	**<.001**	−0.04 ± 0.04	.36	**0.15 ± 0.03**	**<.001**
	(Small)		(Carb rich)		(Circular)	
Treatment	**0.45 ± 0.03**	**<.001**	**0.51 ± 0.04**	**<.001**	**0.45 ± 0.03**	**<.001**
	(Safe)		(Safe)		(Safe)	
Order	**−0.35 ± 0.03**	**<.001**	**−0.35 ± 0.04**	**<.001**	**−0.29 ± 0.04**	**<.001**
	(second)		(second)		(second)	
Trait * treatment	–		**−0.12 ± 0.05**	**.02**	–	
			(Carb rich * safe)			
Trait * order	–		–		**−0.11 ± 0.05**	**.04**
					(Circular * second)	

*Note*: Interactions of seed pairs with perceived predation risk treatment (risky and safe) or randomized order of treatment that individuals underwent in the experiment (first and second) were also included in the model. The models included patch and individual identity as random effects. Shown are estimated fixed effects (*β*), their standard errors (SE) and *p*‐values (*p*). All significant relationships are shown in bold font (ɑ < 0.05) and underlined if still significant with Bonferroni correction (ɑ < 0.016).

**TABLE 4:** Post‐hoc pairwise comparison of the significant interactions of the generalized linear mixed model (GLMM) with the total number of seeds eaten (dependent variable) and interactions of seed pairs (by functional trait) with treatment (safe vs. risky) or order (first or second, that is, randomized order of treatment that individuals underwent in the experiment).

		*β* ± SE	*z*‐ratio	*p*
Seed pair (nutrients)—treatment interactions				
Carb rich—risky	Fat rich—risky	0.04 ± 0.04	0.92	.80
Carb rich—safe	Fat rich—safe	**0.16 ± 0.03**	**4.60**	**<.001**
Carb rich—safe	Carb rich—risky	**−0.39 ± 0.04**	**−9.47**	**<.001**
Fat rich—safe	Fat rich—risky	**−0.51 ± 0.04**	**−12.85**	**<.001**
Seed pair (shape)—order interactions				
Circular—first	Oval—first	**−0.15 ± 0.03**	**−4.77**	**<.001**
Circular—second	Oval—second	−0.04 ± 0.04	−0.98	.76
Oval—second	Oval—first	**0.29 ± 0.04**	**6.96**	**<.001**
Circular—second	Circular—first	**0.40 ± 0.04**	**9.98**	**<.001**

*Note*: Shown are estimated comparison effect (*β*), their standard errors (SE), *z*‐score (*z*‐ratio), and the *p*‐value adjusted with the Tukey method. Effect sizes are reported in a log scale. First column shows the reference pair in all analyses. All significant comparisons are shown in bold.

## 4 DISCUSSION

As predicted, voles in a high predation risk treatment were less active and consumed less resources (higher GUD) than when subjected to lower risk. These patterns are in line with other studies showing higher GUD and less time spent in risky landscapes compared to safe ones (e.g., Eccard et al., 2022; Kotler, 1992; Orrock et al., 2004). Individuals also visited patches less often in the risky treatment, further confirming the pattern that individuals maximized their gains through foraging by minimizing their foraging activity and repeated movements and, therefore, lowering the risk of becoming prey (Dammhahn et al., 2022; Masello et al., 2017). In contrast to our prediction for a central‐place forager, we observed that GUD decreased and cumulative time in a patch increased with higher distance from the shelter, and number of patch visits increased with distance in the safe treatment only. Given that all patches had the same initial gains, this result was unexpected within the context of central‐place foraging theory, as there were no higher quality patches that would justify the repeated travel costs to the most distant patches (Bakker et al., 2005; Nilsson et al., 2020). However, these observations may be due to the small dimensions and fencing of the arena, as foraging closer to the high metal fence could have been perceived as safer than the more exposed patches in the center. We observed in some situations that, when leaving the shelter, bank voles would immediately move to the edges of the arena, and then use that location to go into the patches. While the patches were still separated from the metal fence, future studies should separate patches further from the limits of the arena so the foragers can choose solely to move within the patch array, or increase the size of arenas (e.g., Dammhahn et al., 2022). Albeit the experimental arenas were small and might not fully allow voles to express variation in foraging movement or represent their energy landscapes, we could demonstrate differential movement patterns between safe and risky treatments. Additionally, the safer areas accidentally created in our design (heterogeneous perceived predation risk) further illustrated the decision‐making process of foragers, as this area took priority in their foraging effort distribution despite the increased traveling costs for the forager.

We observed that preference for some functional traits of plant seeds changed depending on perceived risk, even though foragers seemed to prefer larger seeds independently of the risk treatment. This result is in concordance with previous studies, that showed bank voles (Ellingsen et al., 2017; Fischer et al., 2017) and other rodents (e.g., Mortelliti et al., 2019; Wang & Chen, 2009) forage on larger seeds. Under high perceived risk, it is expected that by foraging for shorter periods of time, the individual can be less selective of which seeds they remove, therefore, not express their actual dietary preference (Eccard et al., 2022). When placed in a substrate, it is possible that voles consume the bigger seeds first not by dietary preference but by opportunistic chance, as these larger seeds are not only more visible to the voles (Garb et al., 2000) but also often rise quicker to the top than smaller seeds when the substrate is moved by the digging action of the vole (Gajjar et al., 2021). Therefore, the foraging period is reduced substantially regardless of risk merely by the higher visibility of the seeds. However, bigger seeds also have proportionally more nutrient content per seed than smaller ones, making them more profitable food (Hou et al., 2021; Wang & Corlett, 2017; Wang & Yang, 2014), and this preference might therefore not be affected by varying perceived predation risk (Sivy et al., 2011). A preference for maximizing energetic gains is also evident by the removal of the larger and most nutritious seeds first, while smaller and less nutritious seeds are left in higher densities in the patch, or consumed only after the most preferable seeds are depleted in safer landscapes (Figures 4 and 5; see also Eccard et al., 2022; Ferreira et al., 2022).

In the safe treatment, we found that the foragers expressed a preference for fat‐rich seeds, which also had more caloric content proportionally to the seed size, which was not expressed in the risky treatment. A possible preference for fat‐rich seeds in safer landscapes would allow us to disentangle the importance of two functional traits that are often linked together: size and fat content (Wang & Yang, 2014). Studies on bank voles have previously shown their preference for fat‐rich seeds (Ellingsen et al., 2017; Fischer et al., 2017), especially in pregnant females requiring more nutritional rich food (Eccard & Ylönen, 2006), but this preference is also seen in other rodents (Boone & Mortelliti, 2019; Wang & Chen, 2012). If significant, preference of fat‐rich seeds in safe landscapes could be actual nutrition preference acting, as foragers might feel safe enough to spend more time choosing among seeds and acting on their actual dietary preference, rather than seizing a fast opportunity with the bigger and most visible seeds. This interaction of nutrient type with perceived predation risk treatment also demonstrates that risk landscapes can also affect diversity at GUD (Eccard et al., 2022), as in safer landscapes the carb‐rich seeds are left in higher densities once the forager gives up on feeding. Furthermore, food preference should be taken into account when testing landscapes of fear using artificially provided food sources, as the food item nutritional values can drive part of the results (McMahon et al., 2018). More nutrient types should be investigated in the future, as well as the presence of husk in seeds, as in our study husk, high protein and fiber content were traits only present in the seeds with already high fat content and thus we could not disentangle them.

The third functional trait studied—shape—did not seem to be preferred by foragers among or within treatments. However, foragers seemed to consume more of the circular seeds when tested the first time, while later they showed either no preference for any functional traits and overall ate less seeds than the first time. The seed's shape is a functional trait that is less studied in foragers preference, even though it can influence handling and transportation, two behaviors that are part of the decision making of foraging (Kelrick et al., 1986; Muñoz et al., 2012). The possible preference for circular‐shaped seeds in our study could have been driven by hemp, a large and fat‐rich seed that was always depleted at faster rates regardless of treatment (Figure 5). In comparison to the bigger wheat seed, hemp has a circular shape, relatively smaller mass and is much more caloric in proportion to its size (Table 1), which makes it a seed that is highly nutritious, but also easier to both handle and transport. Other studies have also shown that smaller seeds with high nutritional values can be preferred by foragers due to its lightweight and easiness to transport rather than overall size (Fischer & Türke, 2016; Muñoz & Bonal, 2008), further, a simpler or more circular seed shape can also be preferred due to easier manipulation (Kelrick et al., 1986, Muñoz et al., 2012).

The preference for shape could also be misleading in our results, as overall, individuals showed higher GUD and less active time when submitted to the experiment a second time, regardless of risk treatment. Given its artificial setting, as well as familiarity with previous feeding experiments, it is possible that individuals got habituated to the experiment (Martin & Réale, 2008). One component that we also did not analyze was individual personality, which is known to also influence both foraging behavior under varying predation risk (Dammhahn et al., 2022; Mazza et al., 2019), but also food preference (Boone et al., 2022). Results within individual show that there might be some individually based variation (Figure 4a–f), as some individuals constantly foraged more than others, although its relation to perceived predation risk and/or seed functional traits was not analyzed.

## 5 CONCLUSION

Variation in perceived predation risk impacts the ecology of prey species, affecting their foraging behavior in terms of amount of energy consumed and movement pathways. Prey species navigate in these landscapes of fear carefully to avoid mortality, while still trying to obtain enough resources to survive in the least amount of foraging time. In this study, we observed that the landscape of fear not only affects the amount of food consumed and the active time foraging, but also affects the diet preference of foragers beyond a species' standard preference for certain functional traits. Individuals minimized potential mortality costs by prioritizing safer areas in the landscape, despite moving longer distances, while maximizing the energetic gains by foraging selectively on resources within and among perceived predation risk treatments. Under safer conditions, animals selected seeds for their larger size and fat content, while under risky conditions only a size selection was observed. Thus, risk while foraging affects the remaining seed community diversity. Understanding the variation of behaviors within landscapes of fear helps us to understand the complexity of the non‐consumptive effects of perceived predation risk, and further illuminate the cascading effects of fear on biodiversity.

## AUTHOR CONTRIBUTIONS

**Clara Mendes Ferreira:** Conceptualization (equal); formal analysis (lead); investigation (equal); methodology (equal); writing – original draft (equal); writing – review and editing (equal). **Melanie Dammhahn:** Conceptualization (equal); investigation (equal); methodology (equal); validation (equal); writing – original draft (equal); writing – review and editing (equal). **Jana A. Eccard:** Conceptualization (equal); methodology (equal); validation (equal); writing – original draft (equal); writing – review and editing (equal).

## FUNDING INFORMATION

This study was funded by the German Research Foundation within the BioMove Research Training Group (DFG, GRK 2881/1). During manuscript preparation MD was funded by the German Research Foundation (DA 1377/4‐1). Open access publication was funded by the German Research Foundation (DFG, 491466077).

## Supporting information


Appendix S1

## Data Availability

Datasets used for the analyses are deposited at OSF (DOI: 10.17605/OSF.IO/JQY7D).
