# Supplementary material for: So many choices, so little time: Food preference and movement vary with the landscape of fear
Source: Ecol Evol. 2023 Jul 26;13(7):e10330. doi: 10.1002/ece3.10330 (PMC10372006; doi:10.1002/ece3.10330)
Supplement: Supplementary file 1 — Appendix S1 [file ECE3-13-e10330-s001.docx]

**So many choices, so little time: food preference and movement vary with landscape of fear**

**Supplemental material Appendix 1**

**AnimalTracker analyses**

The plugin AnimalTracker (Gulyás et al. 2016) makes use of AVI video files in ImageJ to track animals in the landscape. Even though our videos spanned for the whole experiment duration of 24h, the whole file was not analyzed due to technological constrains. Since the animals were not active for the entirety of the period, we first divided the videos into smaller video files with the activity bouts. Furthermore, elements in the video could interfere with automatic tracking, so we created a opaque white mask to overlay any element that was not supposed to be tracked (such as the outside of the arena, the water bottle bubbles created by the rodents drinking, and time stamp from the video). This mask was applied to the video using a ffmpeg script (Tomar 2006), also used to convert the video file from MP4 to AVI. The video resolution was also reduced to half (from 1280x720 px to 640x360 px, keeping the frame rate of 25 fps), as tests indicated that this would accelerate the automatic tracking analyses without compromising the quality of the tracking under any conditions. For the safe treatment, we increased the contrast by 0.4x and brightness by 0.15% in ffmpeg to make it easier to track the animal under dark. Following the AnimalTracker instructions, we used the Background substractor with three frames where the animal was either hidden or in different parts of the arena. We used a threshold of 20 for all videos, and the post-processing options were done in the following order: Erode (default parameters), Dilate (default parameters), Close (default parameters), Size filter (10-250). After the automatic tracking was done, we would overview the video to check for errors in tracking. If the program stopped tracking the animal for any reason (could not pick up the animal again or tracked another blob in the arena), we saved the correct part of the tracking and re-did the automatic tracking from the frame where the problem started to occur. Furthermore, we error proofed our final tracking output by tracking sudden changes in coordinates, including: unnatural jumps in coordinates from one frame to another, coordinates that aren’t supposed to exist during a time period (e.g. coordinates outside of the shelter range when the animal was inside the shelter), or repeated coordinates over a span of several frames that could indicate a wrongful tracking of a non-animal blob. If the animal was present, we corrected the coordinates manually or re-did the video analyses to ensure the correct tracking, or if the coordinates were deleted if the animal was not present (thus preventing false tracking points). To calculate the number of visits per patch, we used a raster from the experimental layout from AnimalTracker’s ‘Zone Designer’ module for each experiment run, created a shapefile in qGIS (QGIS.org 2021) and checked which tracking points were within each seed tray.

**References**

Gulyás, M., Bencsik, N., Pusztai, S., Liliom, H., & Schlett, K. (2016). Animaltracker: An imagej-based tracking api to create a customized behaviour analyser program. *Neuroinformatics*, *14*(4), 479–481. <https://doi.org/10.1007/s12021-016-9303-z>

QGIS.org (2021). QGIS Geographic Information System. *QGIS Association*. [http://www.qgis.org](http://www.qgis.org/)

Tomar, S. (2006). Converting video formats with FFmpeg. *Linux Journal, 146*, 10.
